# The Effect of Heat Shock on Myogenic Differentiation of Human Skeletal-Muscle-Derived Mesenchymal Stem/Stromal Cells

**DOI:** 10.3390/cells11203209

**Published:** 2022-10-13

**Authors:** Rokas Mikšiūnas, Siegfried Labeit, Daiva Bironaitė

**Affiliations:** 1 Department of Regenerative Medicine, State Research Institute Centre for Innovative Medicine, Santariskiu 5, LT-08460 Vilnius, Lithuania; 2 Medical Faculty Mannheim, University of Heidelberg, 68169 Mannheim, Germany; 3 Myomedix GmbH, 69151 Neckargemünd, Germany

**Keywords:** skeletal muscle, CD56, heat shock response, myogenic differentiation, mesenchymal stem/stromal cells

## Abstract

Muscle injuries, degenerative diseases and other lesions negatively affect functioning of human skeletomuscular system and thus quality of life. Therefore, the investigation of molecular mechanisms, stimulating myogenic differentiation of primary skeletal-muscle-derived mesenchymal stem/stromal cells (SM-MSCs), is actual and needed. The aim of the present study was to investigate the myogenic differentiation of CD56 (neural cell adhesion molecule, NCAM)-positive and -negative SM-MSCs and their response to the non-cytotoxic heat stimulus. The SM-MSCs were isolated from the post operation muscle tissue, sorted by flow cytometer according to the CD56 biomarker and morphology, surface profile, proliferation and myogenic differentiation has been investigated. Data show that CD56(+) cells were smaller in size, better proliferated and had significantly higher levels of CD146 (MCAM) and CD318 (CDCP1) compared with the CD56(−) cells. At control level, CD56(+) cells significantly more expressed myogenic differentiation markers *MYOD1* and myogenin (*MYOG)* and better differentiated to the myogenic direction. The non-cytotoxic heat stimulus significantly stronger stimulated expression of myogenic markers in CD56(+) than in CD56(−) cells that correlated with the multinucleated cell formation. Data show that regenerative properties of CD56(+) SM-MSCs can be stimulated by an extracellular stimulus and be used as a promising skeletal muscle regenerating tool in vivo.

## 1. Introduction

Physical inactivity, chronic diseases and various pathophysiological factors contribute to the diminished muscle regeneration and poor performance [1]. Therefore, it is important to investigate ways of improvement of skeletal muscle regeneration. The intramuscular regenerative molecular mechanisms are mostly stimulated through the physical activity-induced mechanotransduction, thermal effects, or their combination [2,3]. Both of them increase the intramuscular temperature positively affecting the functioning, recovery and regeneration of skeletal muscle [4]. It was shown that during an intensive exercise, leg muscle temperature of young adult population can increase from 36 °C up to 38 °C improving muscle functioning [5]. Alternatively, an application of external passive heating, both locally or for whole body, can not only reduce pain but also increase muscle strength and stiffness that might be recommended as an exercise mimetic for the limited physical mobility people [6]. However, at molecular level, the muscle tissue regeneration processes are much more complicated and requires activation of multiple surface and intracellular signaling pathways, transcription and paracrine factors, synthesis of various proteins leading to the efficient formation and functioning of myogenic fibers [7]. Although an externally applied thermal stimuli can promote muscle regeneration, the intracellular molecular mechanisms are still not fully clear.

Two main cell groups contribute skeletal muscle repair and regeneration: satellite cells (SC), found underneath the ECM sheet [8,9], and non-satellite cells that guide the stem cells trough the myogenesis [10]. During the initial phases of injury, there is a rapid accumulation of immune cells, that change the environment of the satellite cell niche and activate their proliferation and differentiation to the myoblasts with further generation of new myotubes [11]. The non-satellite cells, such as vessel-associated cells and pericytes [12,13], interstitial cells including fibro/adipogenic progenitors (FAPs) identified by CD15+/PDGFRα+CD56− immunophenotyping [14,15], PW1+/Pax7− cells and muscle side population also support myogenesis [16,17,18]. Since the myogenic progenitors represent approximately 2–7% of skeletal muscle tissue, the alternative sources of cells suitable for the skeletal muscle regenerative studies can be skeletal-muscle-derived mesenchymal stem/stromal cells (SM-MSCs) [19]. However, the SM-MSCs are heterogeneous composed of satellite and non-satellite cells that might have a divergent regenerative potential and different response to an external heat stimuli [19]. Therefore, in this study we have investigated the myogenic potential of total human SM-MSCs population and its CD56(+) and CD56(−) subpopulations.

CD56 (neural cell adhesion molecule, NCAM) is an integral membrane glycoprotein that mediates calcium-independent homophilic cell–cell binding [20]. CD56 is expressed by many cells and tissues, including astrocytes, neurons and glia, cardiac and skeletal muscles, epithelium, immune as well as cancer cells [21]. The CD56, cadherins and other adhesion molecules play a particular role in cell to cell binding, survival and signal transduction [22,23]. It was also shown that mouse myoblasts overexpressing CD56 enhanced myogenesis [24]. In addition, the NCAM/CD56 antigen is the most frequently used biomarker for quiescent as well as activated and proliferating satellite cells [25]. Since the number, quiescence status in the niche and/or activation potential of satellite cell in skeletal muscle is diminishing with the human’s age [26,27], the investigation of molecular mechanisms by which the external stimuli could positively affect an intramuscular stem/stromal cells or their subpopulations is very important in scientific and skeletal muscle regenerative sense.

Thus, the main goal of this work was to investigate the effect of external, non-cytotoxic heat stimulus on the intracellular myogenic differentiation regulating molecular mechanisms of human SM-MSCs-derived, satellite-like CD56(+) and CD56(−) subpopulations. Data show that adult skeletal-muscle-derived SM-MSCs possess an equal number of CD56(−) and CD56(+) cells but with different cellular features, myogenic potential and response to the external heat stimuli in vitro. Data suggest that targeted external thermal stimulation of CD56(+) SM-MSCs subpopulation could further improve skeletal muscle regeneration in vivo.

## 2. Materials and Methods

### 2.1. The Isolation and Cultivation of Total SM-MSCs Population

Human SM-MSCs were isolated from post plastic surgery operation material (muscle pectoralis major) of healthy 30–40-year-old female donors (Bioethics No. 158200-14-741-257). Throughout this study 3–4 SM-MSCs from female donors were used for the experiments. Skeletal muscle specimens were stored on ice and transported to the laboratory for further cell isolation. Muscle specimens were cut into 1 mm^3^ fragments, washed with PBS containing 100 u/mL of streptomycin and 100 u/mL of penicillin (Hyclone, Logan, UT, USA), 1 mg/mL of collagenase I ((Thermo Fisher Scientific, Waltham, MA, USA) was added and incubated at 37 °C for 1 h. After that, DMEM with 4.5 g/L glucose, 10% FBS ((Thermo Fisher Scientific, Waltham, MA, USA) and antibiotics (growth media) was added, and digested fragments were filtered through 100 µM filter. The cell filtrate was resuspended in growth media and seeded on 0.2% gelatin-coated cell flasks (AppliChem GmbH, Darmstadt, DEU). After 3–5 days, the SM-MSCs started to adhere to the precoated surface. The SM-MSC were harvested with trypsin/EDTA (Thermo Fisher Scientific, Waltham, MA, USA) solution and seeded for further cultivation and/or experiments.

### 2.2. Flow Cytometry Analysis

The identification of cell surface and intracellular proteins, and cell sorting was carried out using flow cytometer/sorter BD FACSAria™ II (BD biosciences)).

**For the identification of cell surface proteins**, the SM-MSCs were harvested by trypsin, centrifuged, resuspended in PBS supplemented with 2% BSA (Sigma Aldrich, St. Louis, MO, USA) and incubated on ice for 30 min. After that, the cells were washed with PBS and incubated with antibodies against: integrin beta-1 (CD29-ImmunoglobulinG1-Allophycocyanin (1A-219-T100, Exbio, Praha, Czech Republic)); endoglin (CD105-ImmunoglobulinG1-Allophycocyanin (MHCD10505, Thermo Fisher Scientific, Waltham, MA, USA); thymocyte differentiation antigen 1 (CD90-ImmunoglobulinG1-Fluorescein isothiocyanate (328108, BioLegend, San Diego, CA, USA)); vascular cell adhesion protein 1 (CD106- ImmunoglobulinG1-phycoerythrin (555647, BD Biosciences, San Jose, CA, USA)); ecto-5‘-nucleotidase (CD73-ImmunoglobulinG1-Fluorescein isothiocyanate (561254, BD Biosciences, San Jose, CA, USA)); protein tyrosine phosphatase, receptor type, C (CD45-ImmunoglobulinG2a-Fluorescein isothiocyanate (sc-70686, Santa Cruz Biotechnology, Dallas, TX, USA)); stage-specific embryonic antigen 1 (CD15- ImmunoglobulinG1-Phycoerythrin (555402, BD Biosciences, San Jose, CA, USA)); kinase insert domain receptor (CD309-ImmunoglobulinG1-Phycoerythrin (560494, BD Biosciences, San Jose, CA, USA)); costimulatory protein found on antigen-presenting cells (CD40-ImmunoglobulinG1-Allophycocyanin (555591, BD Biosciences, San Jose, CA, USA)); neural cell adhesion molecule 1 (CD56-ImmunoglobulinG1-Allophycocyanin (555518, BD Biosciences, San Jose, CA, USA)); CUB Domain Containing Protein 1 (CD318-ImmunoglobulinG2b-Phycoerythrin (324006, BioLegend, San Diego, CA, USA)); melanoma cell adhesion molecule (CD146-ImmunoglobulinG1-Phycoerythrin (550315, BD Biosciences, San Jose, CA, USA)); frizzled class receptor 9 (CD349-ImmunoglobulinG1-Phycoerythrin (326706, BioLegend, San Diego, CA, USA)); bone morphogenetic protein receptor type 1A (CD292-ImmunoglobulinG1-Fluorescein isothiocyanate (FAB346F, Bio-Techne, Minneapolis, MN, USA)); protein marker of mesenchymal stem cells (Stro1-Immunoglobulin M-fluorescein isothiocyanate, SC-47733, Santa Cruz Biotechnology, Dallas, TX, USA)) and transmembrane phosphoglycoprotein protein (CD34-ImmunoglobulinG1-Fluorescein isothiocyanate (1F-297-T100, Exbio, Praha, Czech Republic)). After incubation, the cells were washed, centrifuged, resuspended in PBS supplemented with 2% BSA and analyzed with flow cytometer.

**The intracellular protein analysis** was also performed with flow cytometer. Briefly, SM-MSCs were harvested with trypsin/EDTA, washed with PBS and fixed with 4% PFA for 15 minutes at room temperature (RT). After that, cells were suspended in methanol and stored at −20 °C. For the measurement, cells were washed twice with PBS and blocked with 2% BSA in PBS for 30 min. Later, SM-MSC were incubated with primary antibodies against MyoD1 (NB100-5651, Bio-Techne Corporation, Minneapolis, MN, USA), myogenin (ab1835, Abcam, Cambridge, United Kingdom) and Myosin Heavy Chain 1 (ab50967, Abcam, Cambridge, United Kingdom) proteins at RT for 60 min. After the incubation period, cells were washed with PBS and incubated with corresponding secondary Alexa Fluor 488-conjugated antibody (Thermo Fisher Scientific, Waltham, MA, USA). Cells were washed with PBS, measured with BD FACSAria™ II flow cytometer (BD biosciences) and analyzed by DIVA software.

**Cell sorting.** In order to separate the CD56(+) and CD56(−) SM-MSCs subpopulations, the cells were stained with CD56-APC antibody, sorted using flow cytometer and seeded for the further experiments. The percentage of CD56(+) and CD56(−) cells was determined comparing with negative isotype control.

### 2.3. Cell Proliferation Measurement

Proliferation of the cells was performed using a CCK-8 kit (Dojindo, Kumamoto, Japan) according to the manufacturer’s recommendations. Briefly, 1 × 103 of non-sorted (total population) and (CD56(−) and CD56(+) SM-MSCs were seeded into 96-well plate and allowed to adhere for 24 h. The number of cells was determined by adding CCK-8 reagent and measuring absorption at 450 nm with SpectraMax i3 (Molecular Devices, San Jose, CA, USA). In parallel, the number of cells was also calculated in order to eliminate metabolic specificity of measured cells; however, results did not differ from the CCK-8 measurement. The cell proliferation was measured every 24 h for five days.

### 2.4. Differentiation to the MSCs Typical Directions

**For the adipogenic differentiation**, the SM-MSC were seeded into 24-well plate at a density of 30,000 cells/well and adipogenic differentiation was induced by adding adipogenic medium consisting of: DMEM with 1 g/L glucose (Thermo Fisher Scientific, Waltham, MA, USA), 20% FBS (Biochrom GmbH, Berlin, Germany), 1% penicillin/streptomycin (Thermo Fisher Scientific, Waltham, MA, USA), 1 μM dexamethasone and 60 μM indomethacin (Sigma Aldrich, St. Louis, MO, USA), 0.5 μM 3-isobutyl-1-methylxanthine (IBMX) (MyBioSource, San Diego, CA, USA) to fully confluent cells and incubated for 21 day. The level of adipogenic differentiation was identified qualitatively by staining fat lipid droplets with 12 mM Oil Red dye (Carl Roth GmbH, Karlsruhe, Germany) and visualizing with a light microscope. Quantitatively adipogenic differentiation was analyzed by dissolving lipid droplets in 70% isopropanol (Eurochemicals, Vilnius, Lithuania) and spectrophotometrically measuring absorbance at 520 nm.

**For the osteogenic differentiation**, the SM-MSC were seeded into 24-well plate at a density of 20,000 cells/well and osteogenic differentiation medium consisting of: DMEM medium with 4.5 g/L, 1% penicillin/streptomycin (Thermo Fisher Scientific, Waltham, MA, USA), 10% FBS (Biochrom GmbH, Berlin, Germany), 0.1 μM dexamethasone (Sigma Aldrich, St. Louis, MO, USA), 50 μg/mL L-ascorbic acid and 10 mM β-glycerophosphate (Santa Cruz Biotechnology, Dallas, TX, USA) was added to 80% confluent cells and incubated for 21 day. Osteogenic differentiation was evaluated qualitatively by staining the cells with 40 mM Alizarin Red S (Carl Roth GmbH, Karlsruhe, Germany) and evaluating using light microscope and qualitatively by extracting the dye with 10% cetylpyridinium chloride (Sigma Aldrich, St. Louis, MO, USA) and spectrophotometrically measuring absorbance at 562 nm.

**For the chondrogenic differentiation**, the 250,000 cells/15 mL tube of SM-MSC were used for the chondrogenic differentiation. The chondrogenic differentiation was induced by adding a medium consisting of: high glucose (4.5 g/L) DMEM medium, 1% penicillin/streptomycin, 1% insulin–transferrin–selenium (Thermo Fisher Scientific, Waltham, MA, USA), 350 μM L-proline (Carl Roth GmbH, Karlsruhe, Germany), 0.1% dexamethasone, 170 μM ascorbic acid-phosphate (Sigma Aldrich, St. Louis, MO, USA) and 10 ng/mL TGF-β3 (Thermo Fisher Scientific, Waltham, MA, USA) was added. After 21 days of differentiation, the pellets were fixed in 10% formalin and embedded into paraffin. Four-micrometer sections were deparaffinized and stained with toluidine blue three times for 5 min at RT. After this, samples were washed few times with distilled water, one time with 96% ethanol and two times with 100% xylene (all reagents from Sigma Aldrich, St. Louis, MO, USA). Slides with sections were mounted using mounting medium (Sigma Aldrich, St. Louis, MO, USA). The efficiency of chondrogenic differentiation was evaluated microscopically, according to the pink staining of glycosaminoglycan and formation of tight surface layers. The chondrogenic differentiation with TGF-β3 (differentiated) and without TGF-β3 (control) has been compared.

### 2.5. External Heat Stress/Stimulus and Myogenic Differentiation

The cells were seeded into 6 well plates and subjected to the 42 °C heat in water bath for 1 h with subsequent recover under regular growth condition for various periods (0.25 h, 0.5 h, 1 h, 2 h, 4 h, 8 h, 24 h) as described in [28,29]. The efficiency of heat stimulus was evaluated by the upregulation of HSF1 and HSP70/72 (*HSPA1A*/*HSPA1B* genes), as measured by flow cytometer and Western blotting, respectively. Overall, a 2 h recovery period was chosen to induce myogenic differentiation.

For the induction of myogenic differentiation, SM-MSCs were seeded into a 6-well plate at a density of 50,000 cells/well, grown to the 90% confluent and myogenic differentiation medium was added for 7 days. The myogenic differentiation medium consisted of: DMEM with 1 g/L glucose, 1% penicillin/streptomycin and 1%, 2%, 3%, 4% and 5% FBS concentrations were tested in order to find the most effective myogenic differentiation-inducing composition. The most effective myogenic differentiation-inducing medium was with 2% of FBS, which was chosen for further experiments. The differentiation medium was changed every second day. In order to visualize multinucleated cells, the SM-MSCs were washed with PBS, fixed with methanol for 10 min at RT and stained with Giemsa dye (Sigma Aldrich, St. Louis, MO, USA).

To investigate the impact of heat stimulus on the myogenic differentiation, the cells were seeded and cultivated as previously described, subjected to 42 °C heat for 1 h and allowed to recover under regular growth conditions for 2 h before adding myogenic differentiation-inducing medium. The impact of heat stimulus on myogenic differentiation was evaluated by the expression of transcription factors *MYOD1* and *MYOG*, measuring the activity of creatine kinase (CK) and level of structural myotube protein myosin heavy chain 1 (MHC1). The level of MHC1 was visualized immunocytochemically as well.

### 2.6. Gene Expression Analysis

Gene expression analysis was used to determine efficiency of myogenic differentiation markers transcription factors *MYOD1* and *MYOG* with or without heat stimulus. The cells were subjected to the 42 °C heat for 1 h and 2 h of recovery under normal cell growth conditions, the myogenic differentiation-inducing medium (DMEM with 1 g/L glucose, 2% of FBS and antibiotics) (Thermo Fisher Scientific, Waltham, MA, USA)) was added for 1, 3 and 7 days. The cell samples were collected with lysis buffer, RNA was purified using GeneJet RNA purification kit (Thermo Fisher Scientific, Waltham, MA, USA) and RNA was reversely transcribed with the Maxima First Strand cDNA Synthesis Kit (Thermo Fisher Scientific, Waltham, MA, USA) according to the manufacturer’s protocols. Real-time PCR was performed in triplicate using Maxima Probe qPCR Master Mix (2X) (Thermo Fisher Scientific, Waltham, MA, USA) using Stratagene MX-3005P detection instrument (Agilent Technologies, Santa Clara, CA, USA). Following Taqman primers (Thermo Fisher Scientific, Waltham, MA, USA) were used to measure target gene expression: *MYOD1* (Hs00159528_m1), *MYOG* (Hs01072232_m1), *ACTB* (Hs01060665_g1) and *GAPDH* (Hs02786624_g1). The expression of *MYOD1* and *MYOG* genes were normalized to the average of the expression of *ACTB* and *GAPDH* reference genes. Gene expression signals were analyzed using Agilent software (Agilent Technologies, Santa Clara, CA, USA) as follows: gene expression = 2^−ΔCt^ × 10,000,000, where ΔCt = (Cttarget gene-Ctreference gene).

### 2.7. Measurement of Creatine Kinase Activity

Creatine kinase (CK) activity measurement was used to determine efficiency of myogenic differentiation of unsorted, CD56(−) and CD56(+) SM-MSC. The cells were subjected to 42 °C heat stimulus for 1 h, allowed to recover under normal growth conditions for 2 h (the maximum of HSF1 activation) and myogenic differentiation-inducing medium (DMEM with 1 g/L glucose, 2% FBS (Thermo Fisher Scientific, Waltham, MA, USA)) was added to the cells for 1, 3 and 7 days. The samples were scraped with 50 mM potassium phosphate buffer and stored at −80 °C. Creatine kinase activity was measured using Creatine Kinase Activity Assay Kit (MAK116) (Sigma Aldrich, St. Louis, MO, USA) according to the manufacturer’s recommendations. The CK activity measuring kit identifies both brain and muscle CK types. After addition of cell lysate, CK activity was evaluated spectrophotometrically by measuring absorbance at 340 nm with SpectraMax i3 (Molecular Devices, San Jose, CA, USA). Absorption values were normalized to creatine kinase standard curve and expressed as activity units/mL/mg protein. The protein concentration was evaluated using Pierce™ Modified Lowry Protein Assay Kit (Thermo Fisher Scientific, Waltham, MA, USA).

### 2.8. Western Blotting

The cells were washed twice with PBS, samples were lysed in RIPA buffer (150 mM NaCl, 5 mM EDTA, 50 mM Tris, 1% NP-40, 0.5% sodium deoxycholate, 0.1% SDS) supplemented with the complex of protease and phosphatase inhibitors. Protein concentration was determined with Pierce™ Modified Lowry Protein Assay Kit. All samples were equilibrated with 6× sample buffer (DTT 375 mM, Tris-HCl pH 6.8, 6% SDS, 48% glycerol, 9% 2-mercaptoethanol, 0.03% bromphenol blue) and heated at 95 °C for 5 min to denature proteins. SDS-PAGE was performed using 4–12% gradient gels (Thermo Fisher Scientific, Waltham, MA, USA) and proteins were transferred to polyvinylidene fluoride (PVDF) membrane (Thermo Fisher Scientific, Waltham, MA, USA) under standard conditions. Later, the membrane was washed with TBST (Tris buffered saline with 20% Tween 20) and blocked at RT with 3% BSA in TBST for 1 h. Antibodies against: HSP70/72 (genes *HSPA1A*/*HSPA1B*) (ADI-SPA-810-F) (Enzo, New York, NY, USA), HSP27 (G31) #2402 (gene *HSPB1*) (Cell Signaling); HSP90 (C45G5) #4877 (gene *HSP90AA1*) (Cell Signaling), desmin (5332T, Cell Signaling Technology, Danvers, MA, USA), vimentin (Sc-7557, Santa Cruz Biotechnology, Dallas, TX, USA), HSF1 (12972S, Cell Signaling Technology, Danvers, MA, USA), beta-actin (PA1-46296, Thermo Fisher Scientific, Waltham, MA, USA) were used to identify protein levels. Then the membranes were probed with secondary HRP-conjugated antibodies (Thermo Fisher Scientific, Waltham, MA, USA) and chemoluminescence was evaluated using SuperSignal West Pico kit, developer and fixer (Kodak, Rochester, NY, USA).

### 2.9. Imunocitochemistry

Human SM-MSCs were seeded on a round coverslip, the cells were subjected to 42 °C heat stimulus for 1 h and after 2 h of recovery at normal growth conditions, the myogenic differentiation medium (DMEM with 1 g/L of glucose, 2% FBS and antibiotics) (Thermo Fisher Scientific, Waltham, MA, USA) was added for 1, 3 or 7 days. Then, the cells were fixed with 4% PFA solution for 15 min, washed with PBS, permeabilized with 0.1% Triton-X100 at RT and incubated with primary antibody against myosin heavy chain 1 (ab50967, Abcam, Cambridge, United Kingdom) and secondary Alexa Fluor 488-conjugated antibody (Thermo Fisher Scientific, Waltham, MA, USA). 1 mg/mL of diamidine-20-phenylindole dihydrochloride (DAPI) solution was added to stain the cell nuclei. Fluorescence images were acquired with EVOS M7000 microscope.

### 2.10. Statistical Analysis

Data are shown as mean ± standard deviation (SD) and were significant at * *p* ≤ 0.05, ** *p* ≤ 0.01, *** *p* ≤ 0.001 level from not less than 3 repeats of 3 independent experiments and 2–4 cell cultures were investigated. In all experiments, SM-MSCs from 2–4 biopsies were analyzed. Student’s *t*-test was calculated using the Excel and Graphpad Prism 6 programs.

## 3. Results

### 3.1. The Characterization of Total SM-MSCs Population

**Figure 1:**
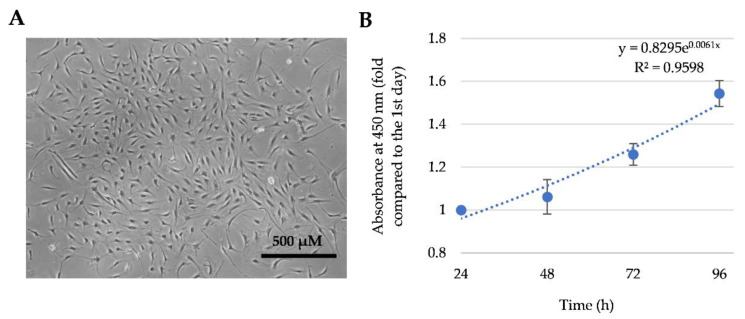
The identification of total SM-MSCs population isolated from human skeletal muscle. (**A**) morphology identified by light microscope; (**B**) proliferation of SM-MSCs. Data are shown as mean ± standard deviation (SD), n = 4 from three independent experiments calculated by an Excel program.

First of all, the morphology and proliferation of isolated total SM-MSCs population have been determined. The cells had spindle shape morphology similar to the other types of primary mesenchymal stromal MSCs (Figure 1A) and showed exponential mode of growth (Figure 1B). The total SM-MSCs population showed some cell size heterogeneity, i.e., some cells were smaller, and some bigger (Figure 1A).

**Figure 2:**
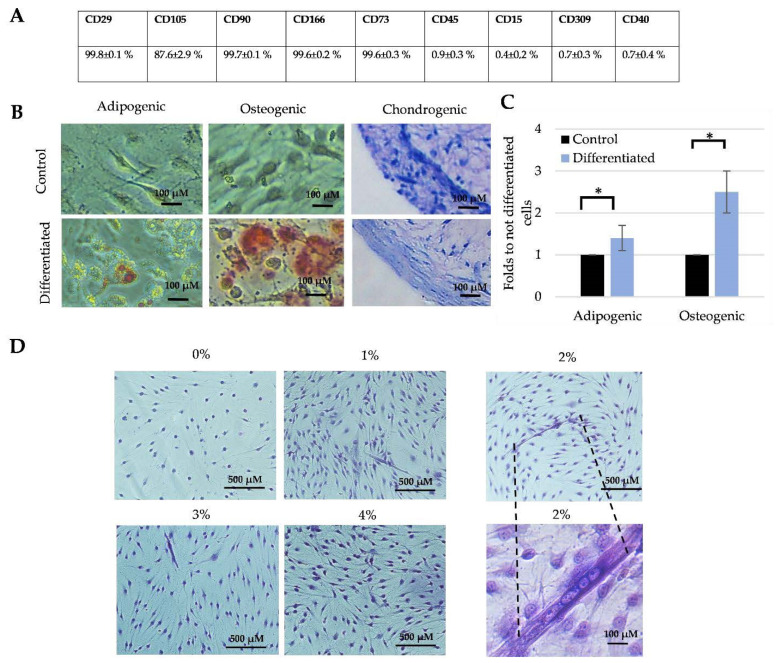
The multipotency of total SM-MSCs population isolated from human skeletal muscle. (**A**) MSCs-typical cell surface marker profile investigated by flow cytometer. (**B**) Qualitative evaluation of adipogenic, osteogenic and chondrogenic differentiation. (**C**) Quantitative evaluation of adipogenic and osteogenic differentiation. (**D**) Myogenic differentiation depending on the FBS concentration. Data are shown as mean ± standard deviation (SD) from not less than three repeats (*n* = 3) and three cell cultures calculated using the Excel software and are significant at * *p* ≤ 0.05 level.

Then the mesenchymal origin (Figure 2A–C) and myogenic differentiation efficiency (D) of total SM-MSCs population have been determined. The human SM-MSCs displayed MCS-typical surface biomarkers which were positive for CD29, CD105, CD90, CD116, CD73 and negative for CD45, CD15, CD309, CD40 surface markers (Figure 2A).

The total SM-MSCs population differentiated weakly from the adipogenic direction (red adipo drops staining with Oil Red) but very intensively differentiated to the osteogenic direction as was qualitatively evaluated by Alizarin Red S staining (red calcium deposits) (Figure 2B). The proteoglycan formation during chondrogenic directions (pink staining with toluidine blue) was also not intensive; however, the tight surface layers demonstrating formation of chondrogenic tissue within 3D chondrogenic differentiation were observed (Figure 2B). In parallel, the quantitative spectrophotometric data of adipogenic and osteogenic differentiation corresponded to the qualitative measurements (Figure 2C).

Then we have investigated the myogenic differentiation conditions for the total SM-MSC culture. The total SM-MSCs population were incubated in DMEM with 1 g/L glucose and 1–4% of FBS for 7 days and cell nuclei were stained with Giemsa dye (Figure 2A–D). The efficiency of myogenic differentiation was evaluated by the formation of multinucleated cells. Data showed that number of multinucleated cells was very low in all cases (Figure 2D). The longest myotubes were formed in DMEM with 1 g/L of glucose and 2% FBS, which was chosen to induce myogenic differentiation. The myogenic differentiation induction with DMEM 1 g/L of glucose and 1% or 2% of horse serum were too harsh and cells did not survive (data are not shown). Myotubes nuclei were stained with Giemsa dye.

So, the total SM-MSCs population showed MCS-typical cell surface biomarkers but weak adipogenic differentiation, which can be related to the skeletal muscle specificity. Since the myogenic differentiation of total MSCs population was also weak, we have tried to stimulate it by an external heat stimulus.

### 3.2. The Impact of Heat Stimulus on the Total SM-MSCs Population

**Figure 3:**
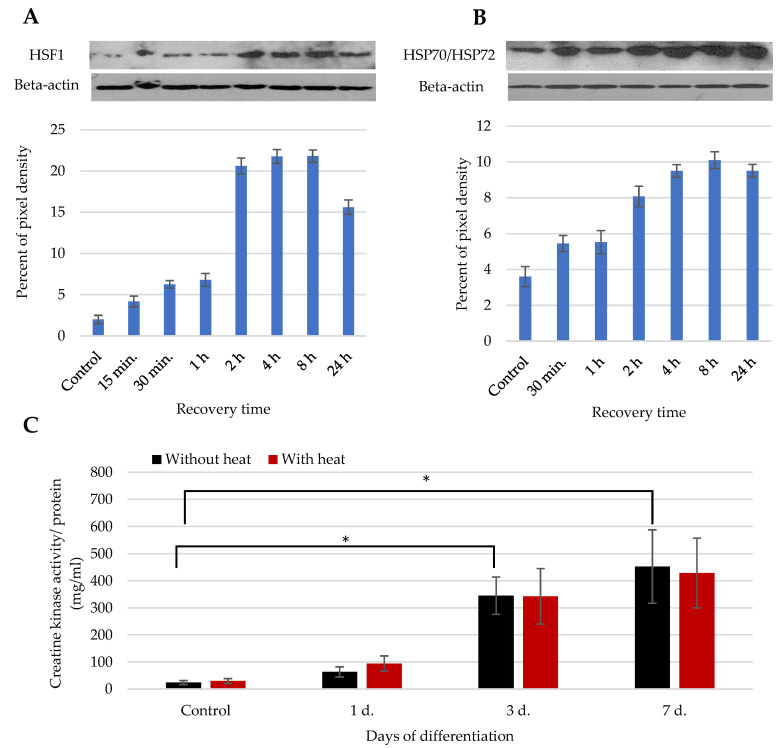
The effect of thermal stimuli on total SM-MSCs population. The level of HSF1 (**A**) (heat shock factor 1) and HSP70/72 (**B**) after the exposure cells to 42 °C heat stimulus for 1 h and different recovery periods (30 min, 1 h, 2 h, 4 h, 8 h, 24 h) under normal growth condition. Representative blots are shown. (**C**) The activity of creatine kinase (CK) in the cells before and after heat stimulus (42 °C for 1 h and 2 h of recovery) for 1, 3 and 7 days in myogenic differentiation medium (2% of FBS in DMEM). Data are shown as mean ± standard deviation (SD) from not less than three repeats (*n* = 3) of three cell cultures calculated using the Excel software and are significant at * *p* ≤ 0.05 level.

The quite low intensity of myogenic differentiation of total SM-MSCs population to encourage the search for solutions. One of the ways can be stimulation of SM-MSCs with an extracellular thermal stimulus (Figure 3). The effect of heat stimulus (at 42 °C for 1 h and different time of recovery under normal growth conditions) on the cells was determined by the induction of heat shock factor (HSF1) and heat shock proteins 70/72 (HSP70/72) (Figure 3A). In this study, antibodies were used to detect both constitutive HSP70 and inducible HSP72 (genes: *HSPA1A*/*HSPA1B*) forms. Thus, changed HSP70/72 level after the heat stimulus is likely attributed to the increase of HSP72. Data showed that level of HSF1 start to increase 30 min after the 1 h heat with the strongest upregulation in 2 h (Figure 3A). The similar tendency has been observed with the heat shock proteins 70/72 (Figure 3B).

Thus, the 2 h recovery period after the heat stimulus has been chosen for the induction of myogenic differentiation and measurement of its marker-creatine kinase (CK). The myogenic differentiation was induced by incubating the cells (with or without heat stimulus) in 1 g/L DMEM with 2% of FBS for 1, 3 and 7 days. Data show that CK activity within myogenic differentiation was steadily increasing; however, heat stimulus did not have a stimulating effect compared with the non-heat-stimulated cells (Figure 3C).

Since heat stimulus did not have a positive effect on CK activity in total SM-MSCs population, it raised an idea that SM-MSCs population might be heterogenic and not all cells equally respond to the external thermal stimulus. Therefore, the total SM-MSCs population was labeled with CD56-APC antibodies (labeling NCAM/CD56-positive satellite-like cells) and sorted by flow cytometer into CD56(+) and CD56(−) subpopulations for the further investigation of myogenic differentiation and impact of heat stimulus on it. 

### 3.3. Sorting and Identification of CD56(−) and CD56(+) SM-MSCs Subpopulations

**Figure 4:**
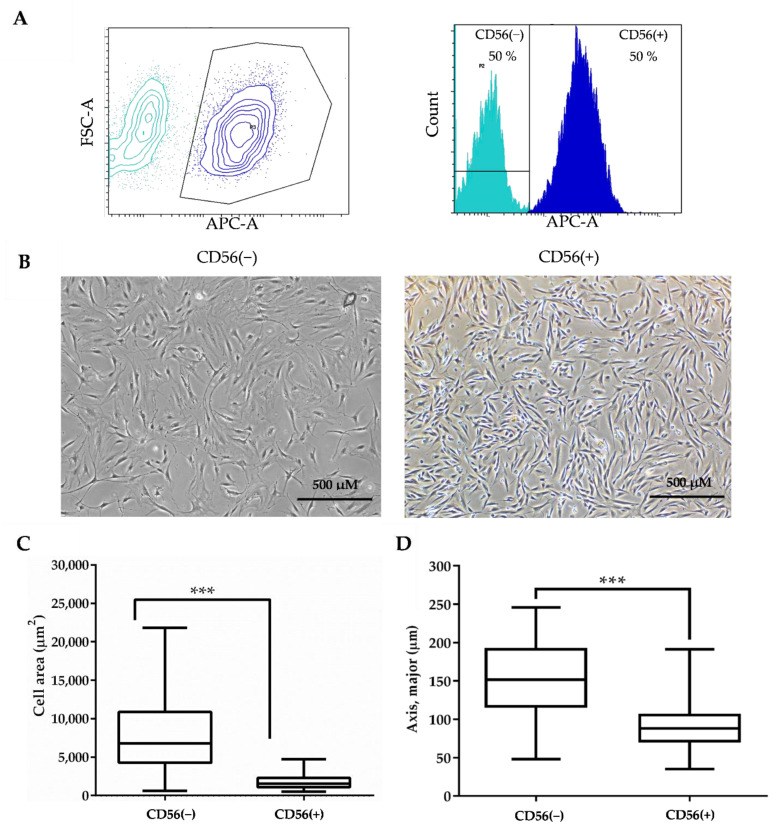
Sorting and morphology analysis of CD56(−) and CD56(+) subpopulations. (**A**) Identification of CD56(−) and CD56(+) subpopulations with BD FACSAria™ flow cytometer. (**B**) Light microscope micrographs of CD56(−) and CD56(+) SM-MSC subpopulations. The evaluation of CD56(−) and CD56(+) cell size according to the cell attachment area (**C**) and longitudinal axis (**D**). Data are shown as mean ± standard deviation (SD). Data are significant at *** *p* ≤ 0.001 level, *n* = 100 from three light microscope micrographs of each cell type. Student’s *t*-test was calculated by the Graphpad Prism 6 program.

The flow cytometry analysis of total SM-MSCs population stained with CD56-APC antibody revealed presence 50% of CD56(+) and 50% of CD56(−) cells (Figure 4A). The light microscope micrographs of sorted cells also showed that CD56(+) cells were smaller in size and grew more compact compared with the CD56(−) ones (Figure 4B). The quantitative analysis of cell size carried out by ImageJ program also confirmed that CD56(+) cells were smaller in size compared to the CD56(−) ones: the same number of the CD56(+) cells occupied almost four-fold less area than the CD56(−) cells (7727 ± 4651 µm^2^ and 1868 ± 990 µm^2^, respectively) (Figure 4C). The longitudinal axis of CD56(+) cells was also almost two-fold less than the CD56(−) cells (153.5 ± 48.53 and 91.24 ± 30.63, respectively) (Figure 4D).

**Figure 5:**
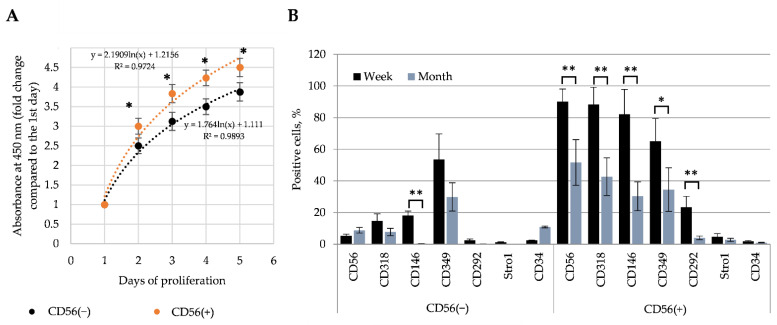
The proliferation and surface markers of CD56(−) and CD56(+) cells. (**A**) Proliferation of human skeletal muscle CD56(−) and CD56(+) subpopulations; (**B**) surface marker profile in CD56(−) and CD56(+) cells after one week and one month of the cell culturing in vitro. Data are shown as mean ± standard deviation (SD) from not less than three repeats (*n* = 3) and three cell cultures calculated using the Excel software and are significant at * *p* ≤ 0.05 and ** *p* ≤ 0.01 levels.

In addition, the proliferation and surface biomarkers of CD56(−) and CD56(+) cells have also been investigated. Data show that CD56(+) cells proliferated better than CD56(−) (Figure 5A). Data suggest that CD56(+) cells might be an activated satellite cells since they proliferated better than CD56(−) cells.

Moreover, various surface markers have been measured in CD56(−) and CD56(+) cells one week and one month after the cell sorting (Figure 5B). The CD56(+) subpopulation, beside the CD56, had a significantly higher levels of myogenic progenitor/satellite cell marker CD318 (transmembrane CUB domain-containing protein 1, CDCP1) and interstitial stem/progenitor cells marker CD146 (melanoma cell adhesion molecule, MCAM) (Figure 5B). The level of CD349 (member of ‘frizzled’ protein family with 7-transmembrane domain; receptors for the activation of Wnt signaling pathway), was around 10% higher than in CD56(−) cells (65 and 54%, respectively). The level of CD292, a bone morphogenetic protein receptor type-1A, was relatively low in both types of the cells: around 23.4% in CD56(+) and only 2.6 percent in CD56(−) cells. The lowest level (only few percent) of the cells were positive for Stro-1, a mesenchymal stromal precursor marker, which suggest that cells are already activated stromal cells (Figure 5B).

The same surface markers have been measured one month after the cell sorting: the levels of almost all surface markers on both cell subpopulations, particularly CD56(+), has been significantly decreased (Figure 5B). Altogether, analysis of cell surface markers suggests that CD56(+) have more myogenic satellite-like and interstitial stem/progenitor cells-typical biomarkers compared with the CD56(−) cells that are decreasing during long-term cell cultivation in vitro. Data also suggest that isolated and/or sorted SM-MSCs should be analyzed as soon as possible.

### 3.4. The Effect of Heat Stimulus on CD56(−) and CD56(+) Cells

**Figure 6:**
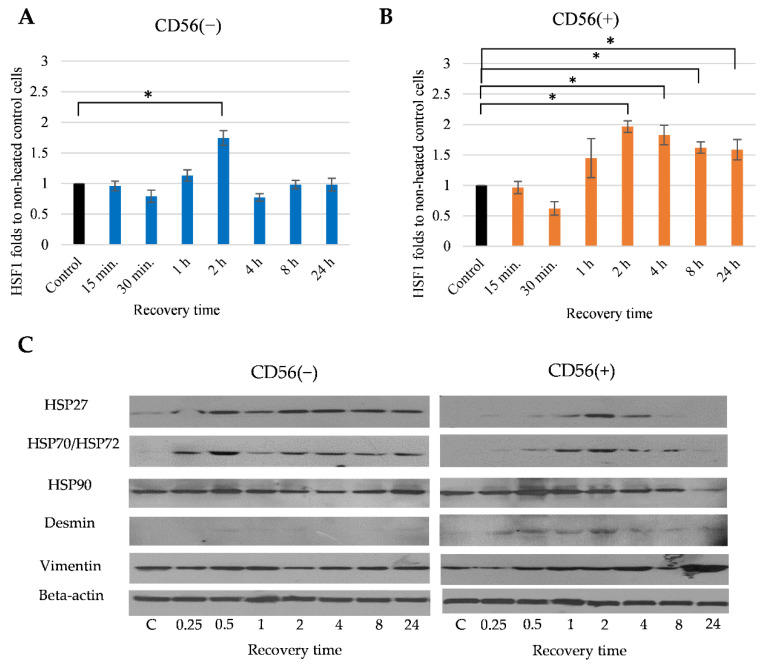
The effect of heat stimulus on the CD56(−) and CD56(+) cells. The level of HSF1 in CD56(−) (**A**) and CD56(+) (**B**) cells estimated by flow cytometer. (**C**). The levels of HSP27, HSP70/72, HSP90, desmin and vimentin in CD56(−) and CD56(+) cells after the 42 °C heat for 1 h and different recover periods (0.25 h, 0.5 h, 1 h, 2 h, 4 h, 8 h, 24 h) under the normal growth conditions. Data are shown as mean ± standard deviation (SD) from not less than three repeats (*n* = 3) and three cell cultures calculated using the Excel software and are significant at * *p* ≤ 0.05. Data show representative WB micrographs of both cell types. The protein level has been equalized using a Lowry protein kit. Control cells were not heat stimulated.

Furthermore, we have investigated how the same conditions of heat stimulus affected HSF1, HSP70/72 and other intracellular proteins in CD56(+) and CD56(−) subpopulations (Figure 6). The level of HSF1 in both subpopulations, similar to the not sorted cells, was significantly increased 2 h after the heat stimulus (Figure 6A,B). However, later on, the level of HSF1 in CD56(−) cells declined, whereas in CD56(+) cells remained almost stable. The data show that both subpopulations responded to the heat stimulus but the HSF1 activation in CD56(−) cells was transient, whereas in CD56(+) the cells were sustained, which might be related to the better activation of myogenic transcription factors and better CD56(+) cell differentiation (Figure 6A,B).

Thermal stimulation in CD56(+) cells also enhanced the levels of other intracellular proteins such as HSP27, HSP70/72 and desmin (myogenic differentiation marker), particularly 2 h after the stimulus, that correlated with the HSF1 activation, while in CD56(−) no significant effect on mentioned proteins was observed (Figure 6C). The level of mesenchymal marker vimentin in CD56(+) was also upregulated particularly 24 h after the heat stimulus. There was no effect of heat stimulus on constitutive HSP90 protein.

Data suggest that thermal stimulation in CD56(+) cells sustainably activated HSF1 and mesenchymal marker vimentin, which might be related to their better myogenic differentiation compared with the CD56(−) cells. This hypothesis has been further investigated measuring expression of myogenic transcription factors *MYOD1* and *MYOG* before and after heat stimulus.

### 3.5. The Effect of Heat Stimulus on the Expression of Myogenic Transcription Factors in CD56(−) and CD56(+) Subpopulations

**Figure 7:**
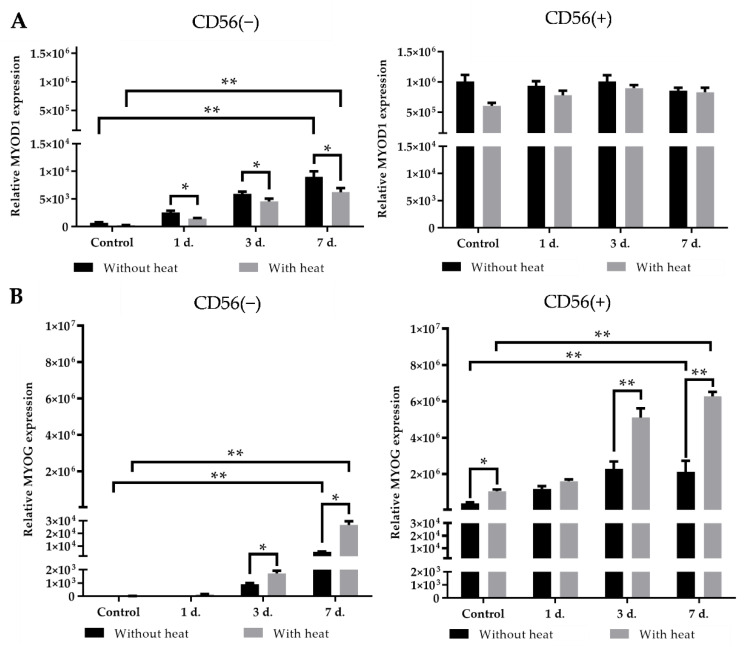
The effect of heat stimulus on the expression of myogenic transcription factors in CD56(−) and CD56(+) subpopulations during 7 days of differentiation. (**A**) Expression of *MYOD1* in CD56(−) and CD56(+) cells with and without heat stimulus; (**B**) expression of *MYOG* in CD56(−) and D56(+) cells with and without heat stimulus. Data are shown as mean ± standard deviation (SD) from not less than three repeats (*n* = 3) and three cell cultures and are significant at * *p* ≤ 0.05 and ** *p* ≤ 0.01 levels calculated using the Graphpad Prism 6 software.

In order to show the effect of heat stimulus on myogenic differentiation of both subpopulations, we have investigated the expression of two transcription factors: myogenic differentiation inducing factor MyoD1 (*MYOD1)* (early myogenic differentiation marker) and myotube generation regulating factor myogenin (*MYOG*) (late myogenic differentiation marker) within 1, 3 and 7 days of cell exposure to the myogenic differentiation-inducing medium (Figure 7).

Data show that at the control level, the expression of *MYOD1* significantly differed between two cell types, i.e., in CD56(+) expression of *MYOD1* was 1700-fold higher than in CD56(−) cells (1,006,260 ± 109,524 and 657 ± 128, respectively) (Figure 7A). Exposing CD56(−) cells to the myogenic differentiation-inducing medium for 1, 3 and 7 days increased the expression of *MYOD1* in CD56(−) cells (from 657±128 at the control level to 9016±1000 at day 7th), whereas in CD56(+), the cells did not (Figure 7A). Moreover, the heat stimulus did not have any positive effect on the expression of *MYOD1* in both cell subpopulations. The explanation might be related to the fact that expression of *MYOD1* in control CD56(+) cells was very high above which stimulus no longer works, whereas in CD56(−) cells *MYOD1* did not respond to the heat stimulus.

The expression of late differentiation marker *MYOG* at control levels was also significantly (around 11 000-fold) higher in CD56(+) cells than in CD56(−) (394,910 ± 50,771 vs. 3.6 ± 0.7) (Figure 7B). The exposure of CD56(−) and CD56(+) cells to the myogenic differentiation-inducing medium for 1, 3, and 7 days increased the expression of *MYOG* in both subpopulations compared to the control cells. The heat stimulus also significantly increased expression of *MYOG* in both subpopulations particularly at 3rd and 7th day of myogenic differentiation. It should be noted that the expression of *MYOG* in heat stimulated CD56(+) cells at the third and seventh day of differentiation was 3000- and 300-fold higher compared with the CD56(−) cells, respectively (Figure 7B). The expression of myogenic transcription factors suggests that heat stimulus stimulates the late myogenic differentiation markers rather than the early ones. In connection with that, we have investigated the activation of other late differentiation markers such as CK and MHC1 at protein levels.

### 3.6. The Effect of Heat Stimulus on the Late Myogenic Differentiation Markers in CD56(−) and CD56(+) Subpopulations

**Figure 8:**
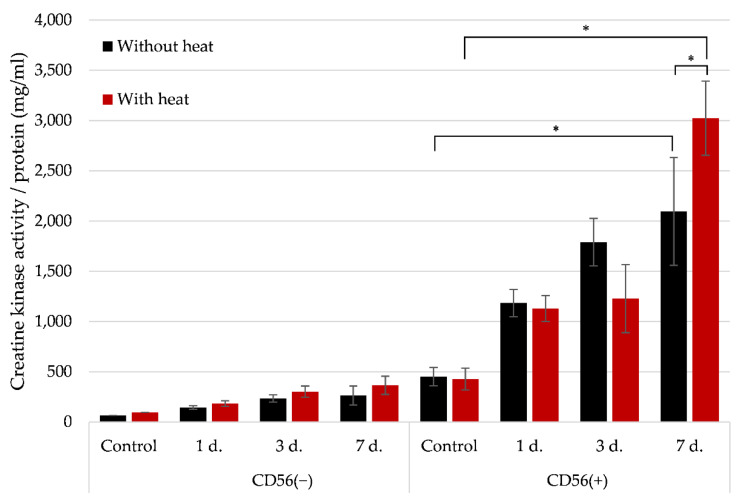
The effect of heat stimulus on creatine kinase activity in CD56(−) and CD56(+) cells. The CD56(−) and CD56(+) cells were heat stimulated (42 °C for 1 h and 2 h of recover) and exposed to the myogenic differentiation medium for 1, 3 and 7 days. The creatine kinase (CK) activity was measured as described in the method part. Data are shown as mean ± standard deviation (SD) and are significant at * *p* ≤ 0.05 from not less than three repeats (*n* = 3) and two cell cultures using the Excel software.

**The creatine kinase (CK)** is known as a cell energetic supplier and one of the late myogenic differentiation markers related to the myotube formation. Used CK kit detects all types of kinases within skeletal muscle, including brain and muscle creatine kinase as well as mitochondrial CK. Muscle creatine kinase (CK-MM) is one of the most abundant proteins in myotubes; therefore, it is safe to assume that changed CK activity is related to the changes to CK-MM [30]. Data show that the CK activity at control cell levels was significantly higher in CD56(+) than in CD56(−) cells (Figure 8). The CK activity within 1, 3, 7 days of myogenic differentiation was significantly (around 8-fold) higher in CD56(+) cells compared with the CD56(−) cells (Figure 8).

The most prominent effect of heat stimulus on CK activity was also observed in CD56(+) cells at 7th day of differentiation, i.e., the CK activity was around 1.5-fold higher compared to the not stimulated cells (2095 ± 419 units/mg/mL proteins before heat stimulus and 3023 ± 302 unit/mg/mL protein after heat stimulus) (Figure 8). It confirms that CD56(+) cells have higher myogenic potential than CD56(−) cells, which can be stimulated by an external heat.

**Figure 9:**
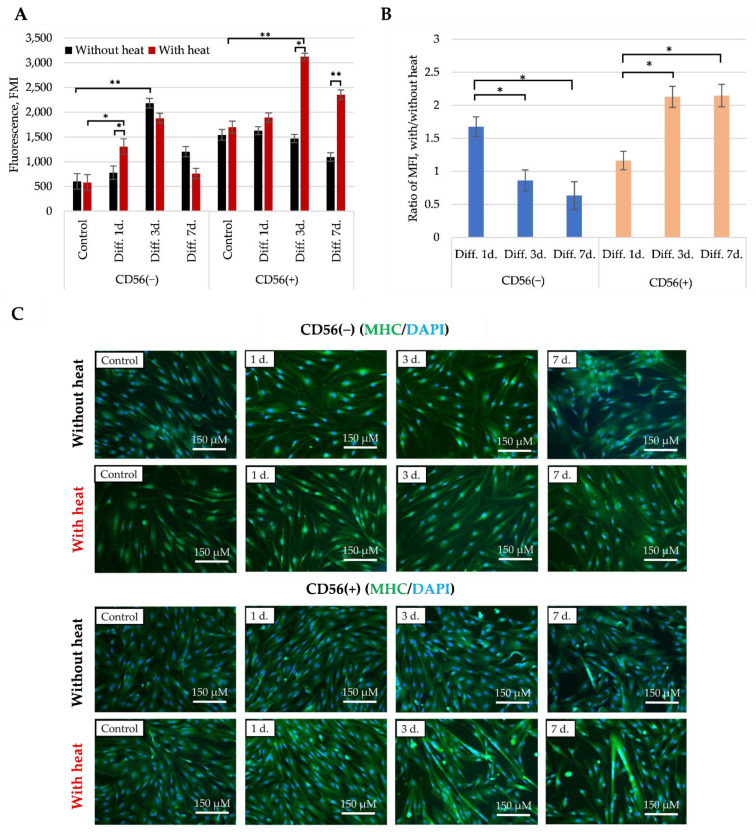
The effect of heat stimulus on the MHC1 level in CD56(−) and CD56(+) cells. The level of MHC1 in CD56(−) and CD56(+) cells was estimated by flow cytometer measuring median fluorescence intensity (MFI) (**A**) and MFI ratio of heat stimulated versus not stimulated cells (**B**). (**C**). The impact of heat stimulus on the MHC1 level and formation of multinucleated cells estimated by fluorescent immunocytochemistry. Data are shown as mean ± standard deviation (SD) and are significant at * *p* ≤ 0.05 and ** *p* ≤ 0.01 levels from not less than three repeats (*n* = 3) and two cell cultures calculated using the Excel software. MHC–myosin-heavy chain 1. Cell fluorescence was visualized with EVOS M7000 microscope.

**The myosin heavy chain 1 (MHC1)** is another late myogenic differentiation identifying biomarker that has been evaluated using flow cytometer (Figure 9A,B) and immunocytochemically (Figure 9C). Surprisingly, the level of MHC1 in both subpopulations was increasing during 1 or 3 days of myogenic differentiation compared to the not stimulated cells but declined at day 7 (Figure 9A). The heat stimulus significantly upregulated MHC1 in CD56(+) cells particularly at the third and seventh days of differentiation compared with the non-stimulated cells (Figure 9B). The heat stimulus did not stimulate the CK activity in CD56(−) cells. Moreover, the immunocytochemical fluorescence confirmed more efficient formation of multinucleated cells/myotubes in heat stimulated CD56(+) cells than in CD56(−) cells at the third and seventh days of differentiation (Figure 9C).

Data suggest that external heat stimulus-induced myogenic differentiation is not a quick process and requires a certain time. It might explain why an external thermal stimulus did not activate an early myogenic differentiation factor *MYOD1* but rather stimulated late differentiation biomarkers such as transcription factor *MYOG1*, CK and MHC1.

## 4. Discussion

Muscle tissue is a dynamic and actively regenerating tissue with remarkable ability to respond to various types of environmental stimuli necessary for the stem cell activation, muscle healing and regeneration processes [31]. Among various types of muscle regenerating cells, the most important were role play satellite cells [8,32]. In response to injury, mechanical or other stimuli, the satellite cells are activated and become myoblasts that further assemble together to form new myofibers [33]. To overcome the limited number of quiescent satellite cells and/or their senescence, the adult human skeletal-muscle-derived primary mesenchymal stem/stromal cells (SM-MSCs) are under intensive investigation [19]. In this study investigated human SM-MSCs had the MSCs-typical cell surface markers, but the differentiation potential was not equally strong to that which might be related to the skeletal muscle specificity. In addition, the SM-derived MSCs were shown to have higher myogenic commitment in vitro and better skeletal muscle regenerating properties in vivo compared with the other types of primary stromal cells [34]. However, various types of adult MSCs, as well as SM-MSCs, are a heterogeneous population not equally responding to the stimulus that are necessary to re-enter cell cycle, myogenic differentiation, muscle growth and regeneration [35]. Since satellite cells are the main cells regenerating skeletal muscle, their typical biomarkers are still under investigation; the presence of biomarkers strongly depends on muscle type, embryonic development, fiber association, postnatal stage and many other cellular and environmental factors [32]. Therefore, in this study we have investigated the myogenic differentiation of CD56-positive SM-MSCs that can be named as activated satellite-like cells and their response to the external heat stimuli.

For a long time, the impact of CD56, a neural adhesion molecule (NCAM), has been investigated only as a marker of human neural stem cells participating mainly in neuro differentiation and/or regeneration processes [36]. Later on, CD56 was shown to be expressed on activated myogenic satellite cells named myoblasts [37,38,39], while CD56(−) are more fibroblasts-like cells expressing TE-7 marker and differentiating to the adipogenic direction [40]. The craniofacial CD56(+) muscle-derived cells was shown to have biomarkers similar to other types of MSCs and multipotential differentiation character [41]. In addition, CD56 expressed on various types of MSCs influenced their differentiation potential [42]. Data of this study showed that isolated CD56(+) cells were smaller in size and better proliferated than CD56(−) cells. Beside the MSCs biomarkers, the CD56(+) SM-MSCs also had significantly higher levels of another satellite cell-typical biomarkers compared to the CD56(−) cells: CD318, known as CUB domain-containing transmembrane protein 1 (CDCP1) (90–95% vs. 15%) and muscle side population biomarker CD146 (melanoma cells adhesion molecule, MCAM) (90–95% vs. 20%) [43,44]. The level of CD349, a Frizzled-9 protein and receptor for Wnt signaling proteins, was also higher in CD56(+) than in CD56(−) cells (65% vs. 54%). It was shown that CD318-positive myogenic progenitors are important regulators of myogenic differentiation and muscle regeneration [43]. The CD146 was also detected on skeletal muscle progenitor cells (SMPC) derived from pluripotent stem cells (PSCs) [45] and was shown to be associated with multipotency of MSCs and a greater differential potential [46,47]. In addition, the CD146 is also highly expressed on the vascular walls and associated with better blood vessel functioning [48]. Since striated muscle functioning, healing and regeneration requires new blood vessels, the CD146(+)/CD56(+) cells could help to promote neovascularization of striated muscles [49].

The myogenesis is generally described as a tightly orchestrated process that is controlled by the helix–loop–helix transcription factors (MRF) such as Myf-5, MRF4, MyoD1 and myogenin regulating myogenic differentiation [50]. The quiescent satellite cells usually express high level of transcription factor Pax7, which is lost after the injury or other stimuli due to the satellite cells activation and/or proliferation [51]. Paired box transcription factor Pax7 appears to regulate the balance between quiescent, activated or differentiated satellite cells [52]. The Pax7(−) and MyoD1(+) were shown to be expressed in proliferating satellite cells; therefore, the MyoD1 is an early myogenic differentiation regulating transcription factor, whereas myogenin mainly regulates terminal cell commitment and myotube formation [25,53]. During the initiation of myogenic differentiation, committed or activated satellite cells become myoblasts expressing *MyoD1* and *MYF5.* The further myotube formation is related to the activation of MRF transcription factor myogenin, fusion of the myoblasts and new muscle fiber generation [51,54]. Which of the MRF transcription factors is most important for the myogenic differentiation, however, is still not clear. It was shown that mice lacking both Myf5 and MyoD1 are totally devoid of myoblasts and myofibers, whereas the absence of myogenin is impairing myofibre formation [41,55,56]. In addition, the intensive and long-term training can activate myogenic differentiation-related MRFs transcription factors leading to the improved muscle regenerative capacity [57]. Data of this study show that at a control level, the CD56(+) cells expressed significantly higher levels of *MYOD1* and *MYOG* compared with the CD56(−) cells, showing their higher commitment to the myogenic differentiation. The external heat stimulus activated expression of late myogenic differentiation transcription factor *MYOG,* not a differentiation initiator *MYOD1*, particularly in CD56(+) cells at the third and seventh days of myogenic differentiation.

In addition, the non-satellite cells also can support myogenic differentiation and was shown to be able to differentiate to the myogenic direction or indirectly support muscle regenerative processes [18,58]. The CD56(−) cells was suggested to contain more fibroblast-like features than CD56(+) cells [15,40]. However, it was shown that fibroblasts are essential for the extracellular matrix synthesis (ECM), which supports muscle elasticity particularly during skeletal muscle strain and exercise [59]. There are also findings indicating that load-induced strengthening of skeletal muscle ECM plays an important role in preventing future muscle damages [60]. Data of this study show that CD56(−) cells might be somewhat involved in myogenic differentiation by showing slight upregulation of transcription factors and late differentiation markers. However, the indirect participation of CD56(−) SM-MSCs in myogenic differentiation by secretion of paracrine factors needs more detailed investigation.

During the final stage of myogenic differentiation, myoblasts fuse into myotubes and fibres participating in muscle contraction, which is a high-energy-demanding process [61]. The morphological myotube formation-related myosin heavy chain 1 and energy supplier muscle creatine kinase are the biomarkers of middle to late stages of myogenic differentiation, respectively [62,63]. It was shown that CK is essential for the highly active tissues such as the brain and muscles to store phosphocreatine and to use it for the rapid ATP production [64]. There are two types of CK: the brain CK is expressed in the brain, heart and smooth muscle, whereas muscle CK is mainly found in heart and skeletal muscle [65]. In addition, the skeletal muscle fibers express mitochondrial CK, however the muscle CK form is one the most abundant proteins in skeletal muscle fibers and likely has a largest impact on CK level in vitro [30]. Beside the proteins energetically helping myotube formation, the intermediate filament proteins such as vimentin or desmin can also respond to the heat stimuli providing antiapoptotic activity, regulating organelle functioning and serving as a scaffold for the binding of other muscle-contraction-regulating proteins [66]. Desmin is a muscle-specific protein of type III intermediate filament found in multiple types of muscle, including cardiac, smooth and skeletal muscle and was shown to be able to react to the environmental changes and stimuli [67]. Data of this study show that external heat stimulus, similar to the transcription factor *MYOG*, activates both biomarkers of late differentiation stage CK and MHC1 in CD56(+), particularly within the 3–7 days of differentiation.

The heat shock response (HSR) is a classical cell and tissue response to the external thermal stimuli inducing adaptation to changed environment conditions. The HSR also plays multiple roles in stem cell development and aging [68]. After the exposure of cells to a non-toxic heat, the heat shock factor 1 (HSF1) becomes trimeric and binds to the genomic heat shock element (HSE), resulting in the induction of heat shock proteins and better myogenic cell survival [28,29,69]. In addition, there is evidence that heat-shock-induced proteins, particularly Hsp70/72, protected satellite cells and preserved their total amount in mice muscle [70]. On the other hand, the level of HSF1 was shown to increase during myogenic differentiation of mouse C2C12 myoblasts, suggesting its involvement in muscle regeneration [71]. The non-cytotoxic heat stimulus, beside the Hsp proteins, also activates various signalling pathways such as ERK, AKT and other improving cell functioning [29,72]. It is likely that heat stimulus is a complex process directly and indirectly activating transcription factors, proteins, paracrine factors and signalling pathways in one way or another, promoting myogenic differentiation. In this study we have investigated intracellular changes of CD56(+) and CD56(−) cells during myogenic differentiation in vitro revealing CD56(+) cells having a higher myogenic potential than CD56(−) cells, which can be extracellularly heat-stimulated. The skeletal-muscle-derived MSCs are a heterogenic population, and a higher number of CD56-positive cells located in the skeletal muscle could improve its functioning in vivo.

## 5. Conclusions

The skeletal-muscle-derived CD56(+) MSCs seems to be activated satellite-like cells, while CD56(−) cell are more fibroblast-like cells. The CD56(+) had slightly different morphology, better proliferated and were more sensitive to the non-cytotoxic heat stimulus than CD56(−) cells. The control CD56(+) cells had a higher myogenic potential than CD56(−) cells, which has been confirmed by the higher expression of early and late myogenic differentiation regulating transcription factors *MYOD1* and *MYOG*, respectively, and the late differentiation markers CK and MHC1. The CD56(+) cells were also better differentiated to the myogenic direction, which was significantly stimulated by heat stimulus.

Data suggest that CD56(+) and CD56(−) subpopulations have different myogenic potential, and muscle cells with higher levels of CD56 can be more responsive to the external stimulating factors improving skeletal muscle functioning. Moreover, the ability to increase the CD56 level in primary skeletal muscle stromal cells in vivo could open new ways of skeletal muscle regeneration. Further detailed investigations in this field are needed.

## 6. Patents

This section is not mandatory but may be added if there are patents resulting from the work reported in this manuscript.

## Data Availability

Data are contained within the article.
